# Implementation of microsatellite instability testing for the assessment of solid tumors in clinical practice

**DOI:** 10.1002/cam4.5569

**Published:** 2022-12-26

**Authors:** Izuma Nakayama, Eiji Shinozaki, Hiroshi Kawachi, Takashi Sasaki, Mayu Yunokawa, Junichi Tomomatsu, Takeshi Yuasa, Satoru Kitazono, Kokoro Kobayashi, Keiko Hayakawa, Arisa Ueki, Shunji Takahashi, Kensei Yamaguchi

**Affiliations:** ^1^ Department of Gastroenterological Chemotherapy Cancer Institute Hospital of the Japanese Foundation for Cancer Research Tokyo Japan; ^2^ Department of Pathology Cancer Institute Hospital of the Japanese Foundation for Cancer Research Tokyo Japan; ^3^ Department of Hepato‐Biliary‐Pancreatic Medicine Cancer Institute Hospital of the Japanese Foundation for Cancer Research Tokyo Japan; ^4^ Department of Medical Oncology Cancer Institute Hospital of the Japanese Foundation for Cancer Research Tokyo Japan; ^5^ Department of Gynecologic Oncology Cancer Institute Hospital of the Japanese Foundation for Cancer Research Tokyo Japan; ^6^ Division of Genitourinary Chemotherapy Cancer Institute Hospital of the Japanese Foundation for Cancer Research Tokyo Japan; ^7^ Department of Thoracic Medical Oncology Cancer Institute Hospital of the Japanese Foundation for Cancer Research Tokyo Japan; ^8^ Department of Breast Medical Oncology Cancer Institute Hospital of the Japanese Foundation for Cancer Research Tokyo Japan; ^9^ Department of Orthopedic Oncology Cancer Institute Hospital of the Japanese Foundation for Cancer Research Tokyo Japan; ^10^ Department of Clinical Genetic Oncology Cancer Institute Hospital of the Japanese Foundation for Cancer Research Tokyo Japan

**Keywords:** DNA mismatch repair, genetic testing, immune checkpoint inhibitors, precision medicine

## Abstract

**Background:**

In Japan, microsatellite instability (MSI) testing for solid tumors was introduced in clinical practice in December 2018. Although immune checkpoint inhibitors (ICIs) are established standards of care for patients with MSI‐high tumors, the status of implementing MSI testing in clinical practice remains unclear.

**Methods:**

We retrospectively reviewed the medical records of patients with solid tumors who underwent MSI testing between January 2019 and December 2020 at our institution.

**Results:**

In total, 1,052 MSI tests were performed in 1,047 patients. Regardless of specimen volume and condition, the MSI status was successfully determined in 1,041 (99.0%) tests, encompassing 27 tumor types (microsatellite stable [MSS] or MSI‐low: *n* = 991 [95.2%] and MSI‐high: *n* = 50 [4.8%]). Patients whose specimens were fixed with 20% neutral buffered formalin (NBF) and who had specimens with prolonged storage (98.4% and 95.4%) showed lower success rates than those whose specimens were fixed with 10% NBF and who had specimens with nonprolonged storage (100.0% and 99.6%), respectively. The prolonged turnaround time (TAT) in MSI‐high cases (median TAT: 24 days) was a critical issue that directly resulted in treatment delay. Of the 50 patients with MSI‐high tumors, 24 (48.0%) received ICIs and 34 (68.0%) were referred to the Department of Clinical Genetic Oncology where 6 (12.0%) patients were diagnosed with Lynch syndrome.

**Conclusions:**

MSI testing was successfully performed for various types of tumors and specimens in clinical practice. Our study results identified certain issues associated with the clinical implementation of MSI testing, including optimal specimen selection, extended TAT in MSI‐high cases, and awareness of hereditary tumors.

## Graphical abstract

More than 1000 real‐world experiences have refined the versatility and reliability of MSI testing. However, turnaround time can be influenced by sample quality and MSI status. Furthermore, awareness about the importance of hereditary tumors by clinicians is a necessary aspect of future growth in precision oncology.

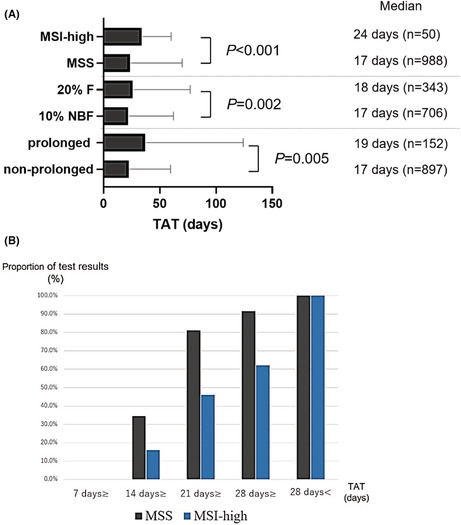

## 1 INTRODUCTION

Immune checkpoint inhibitors (ICIs) were introduced in clinical practice in the 2010s for treating various tumors.^1^ Currently, the combination of immuno‐oncology and ICIs has shown durable responses and manageable toxicities and is globally recognized as an established therapeutic strategy in clinical oncology.^2^, ^3^ A solid tumor with high microsatellite instability (MSI) has deficient DNA mismatch repair (dMMR), which causes hypermutation and produces mutation‐generated neoantigens that elicit immune cell responses.^4^, ^5^ Therefore, theoretically, patients with MSI‐high solid tumors are considered good candidates for ICI treatment.^6^, ^7^ In the KEYNOTE (KN) 016 study, the effectiveness of pembrolizumab, an anti‐programmed cell death 1 (PD‐1) inhibitor, was first demonstrated in patients with dMMR; however, it was ineffective in those with proficient MMR (pMMR).^8^ Subsequent clinical trials in patients with dMMR/MSI‐high tumors have improved the detection of ICI responders regardless of their origin.^9^, ^10^, ^11^, ^12^ In November 2018, the Promega MSI Analysis System was approved as an in vitro diagnostic test to identify MSI‐high solid tumors and determine their suitability for pembrolizumab treatment in Japan.^13^, ^14^ Thus, MSI testing is increasingly being conducted to determine the suitability of patients for ICI treatment, screen for Lynch syndrome, and consider the use of adjuvant chemotherapy for colorectal cancer (CRC) following curative resection. However, several issues associated with the implementation of MSI testing in clinical practice remain unclear, including the turnaround time (TAT), availability of sufficient tissue from small specimens, DNA degradation due to prolonged storage, and collaboration with genetic counselors. Herein, we evaluated our real‐world experience with MSI testing to identify issues associated with its implementation in clinical practice, thus enabling further advancement of precision oncology.

## 2 MATERIALS AND METHODS

### 2.1 Study design and patients

We retrospectively reviewed the medical records of patients with solid tumors who underwent MSI testing between January 2019 and December 2020 at our institution. We enrolled patients who met the following inclusion criteria^1^: patients with solid tumors,^2^ those who underwent polymerase chain reaction (PCR)‐based MSI testing at our institution, and^3^ those who provided written informed consent for MSI testing. We followed the treatment schedule as specified in previous pivotal clinical trials.^9^, ^10^, ^11^ This study was approved by the ethics committee of the Cancer Institute Hospital of the Japanese Foundation for Cancer Research (JFCR) in Tokyo, Japan (approval no. 2020–1229), and was conducted in accordance with the tenets of the Declaration of Helsinki (1964) and its later amendments. Considering the retrospective nature of this study and the option for patients to opt out, the need for informed consent was waived.

### 2.2 MSI testing procedure

The actual MSI testing was outsourced to an inspection company (LSI Medience corporation). Pathologists selected optimal specimens with adequate tumor cells. At our institution, since January 2018, only 10% neutral buffered formalin (NBF) was used to fix tissue biopsy specimens, whereas 20% NBF was used in other cases. After extracting DNA from the formalin‐fixed paraffin‐embedded tissue specimens, MSI testing was conducted via a PCR‐based MSI analysis system (FALCO Biosystems Ltd.) using five quasimonomorphic mononucleotide repeat markers (NR‐21, BAT‐25, MONO‐27, NR‐24, and BAT‐26). These mononucleotide markers have few germline variant alleles; therefore, the MSI status could be determined based on the quasimonomorphic variation range (QMVR) without using normal controls.^15^ However, some cases required normal controls to identify the MSI status. MSI status was classified as MSI‐high and microsatellite stable (MSS). MSI‐high was defined as the detection of the size shift in the PCR band outside QMVR in two or more of the five markers, whereas MSS was defined as the detection of one or no unstable marker.^13^ Samples with weak fluorescence intensity after amplification indicated DNA degradation and were retested using a higher number of PCR cycles.

### 2.3 Indication for MSI testing and ICI

PCR MSI testing, not MMR–immunohistochemistry (IHC) analysis, is the only diagnostic method for determining ICI indication for MSI‐high cases in Japan. In addition, PCR‐based MSI testing has been approved as a screening tool for Lynch syndrome. As MSI testing is occasionally performed in patients with CRC before adjuvant chemotherapy, early stage patients with MSI‐high tumors may not receive ICI.

### 2.4 Statistical analyses

Statistical analyses were performed using Fisher's exact test for categorical data and Mann–Whitney test for continuous data. A *p*‐value of <0.05 was considered statistically significant for all analyses. We used Kaplan–Meier survival curves to calculate overall survival (OS) and progression‐free survival (PFS). OS was defined as the time from the start of chemotherapy to the latest follow‐up or death. PFS was defined as the time from the start of chemotherapy to the first day of disease progression or death. The cutoff date for survival and progression was October 30, 2021. For patients with target lesions, the objective response rate (ORR) and disease control rate (DCR) were calculated according to the Response Evaluation Criteria in Solid Tumors guidelines (version 1.1).^16^ All statistical analyses were performed using the graphical user interface for R (The R Foundation for Statistical Computing) and GraphPad Prism v 9.0 for Windows (GraphPad Software Inc.).

## 3 RESULTS

### 3.1 Feasibility of MSI testing

**FIGURE 1:**
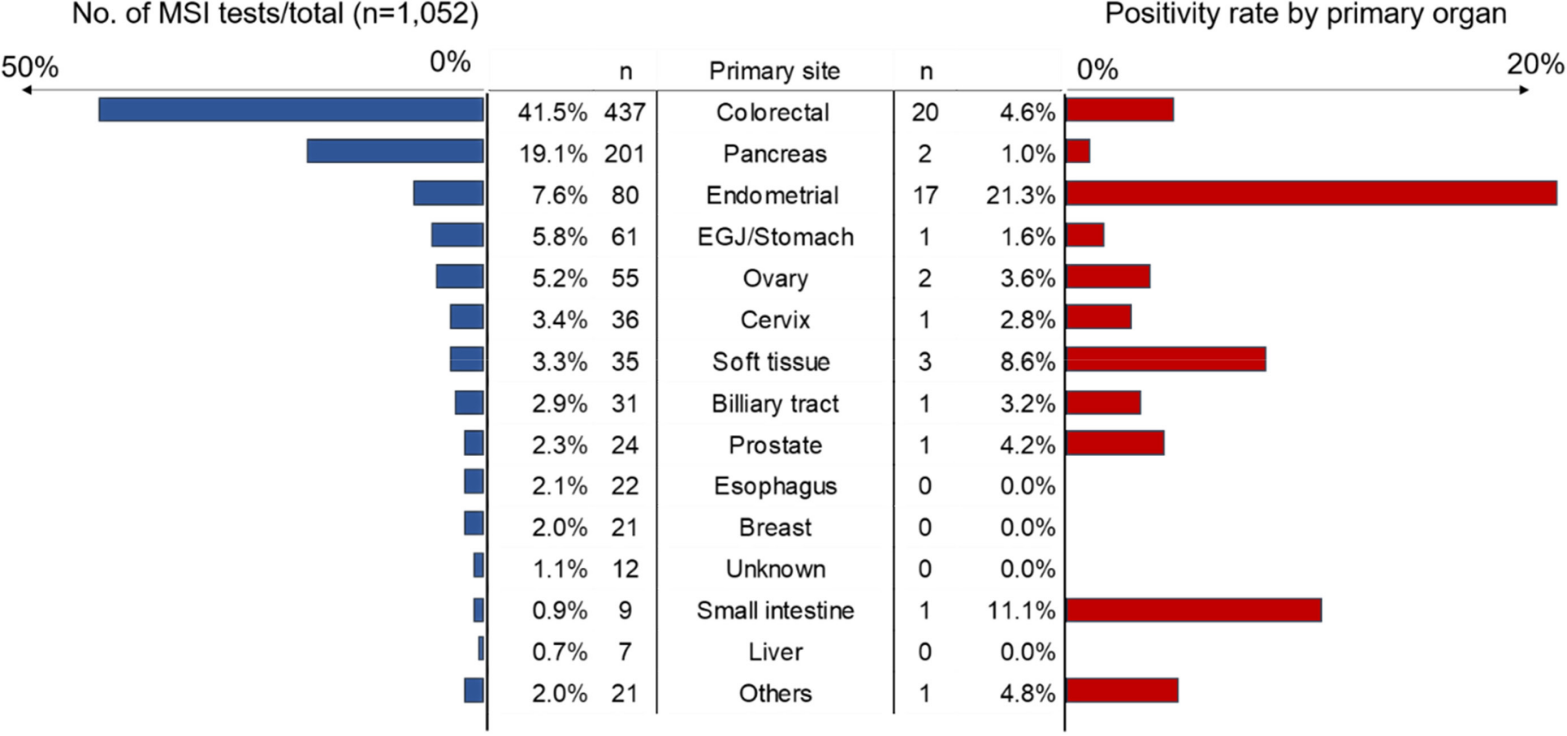
Distribution of primary tumors. The blue bars indicate the percentage of patients who underwent MSI testing as a percentage of the total number (*n* = 1052), according to the primary site. The red bars indicate the percentage of MSI‐high cases per tumor site. MSI, Microsatellite instability.

Between January 2019 and December 2020, 1052 consecutive MSI tests were conducted in 1047 patients with solid tumors at the Cancer Institute Hospital of JFCR. Among them, five patients underwent MSI testing twice, including two patients with two synchronous primary cancers, two with primary and metastatic cancers, and one whose specimen was unsuitable for initial testing. In total, we assessed 27 different types of solid tumors. Patients with CRC accounted for approximately 40% (*n* = 437) of the cohort, of which only 4.6% (*n* = 20) was MSI‐high. Patients with MSI‐high endometrial cancer showed the highest proportion (*n* = 17, 21.3%) (Figure 1). None of the patients who underwent MSI testing twice had MSI‐high tumors. Table 1 presents the success rates of MSI testing. The MSI status could be determined in 1041 of 1052 cases, and the overall success rate of MSI testing was 99.0% (95% confidence interval [CI]: 98.0–100.0). The detection rate of MSI‐high cases was 4.7% (*n* = 50) in the entire cohort. Patients with surgically or endoscopically resected and biopsy specimens, including those collected via fine‐needle aspiration, underwent successful MSI testing (98.6% and 99.5%, respectively). However, based on specimen conditions, some differences were noted in the success rates of MSI testing. Patients whose specimens were fixed with 20% NBF showed a lower success rate (98.4%; 95% CI: 97.2–99.2) than those whose specimens were fixed using 10% NBF (100.0%; 95% CI: 99.1–100.0). In addition, patients who had specimens with prolonged storage (>36 months) showed a significantly lower success rate (95.4%; 95% CI: 90.7–98.1) than those who had specimens with nonprolonged storage (99.6%; 95% CI: 98.9–99.9) (Table 1). For patients whose specimens were fixed with 20% formalin, a statistically significant difference was noted in the success rates of MSI testing between specimens with prolonged and nonprolonged storage (95.4% [95% CI: 90.7–98.1] vs. 99.3% [95% CI: 98.2–99.8]; *p* = 0.0028). However, for patients who had specimens with nonprolonged storage, no significant difference was observed in the success rates of MSI testing between specimens fixed with 10% and 20% NBF.

**TABLE 1:** Success rate of MSI testing

	Total	Success rate	95% CI	*p*‐Value	Determinable		Undeterminable
					MSS	MSI‐high	
All, *n* (%)	1052 (100.0)	99.0%	98.0–100.0		991 (94.2)	50 (4.7)	11 (1.0)
Specimen material							
Surgical, *n* (%)	666 (63.3)	98.6%	97.5–99.4	0.346	615	42	9
Nonsurgical, *n* (%)	386 (36.7)	99.5%	98.1–99.4		376	8	2
Fixated formalin concentration							
10% NBF	344 (32.7)	100.0%	99.1–100.0	0.020	337	7	0
20% NBF	708 (67.3)	98.4%	97.2–99.2		654	43	11
Interval between specimen collection and testing							
Specimens with nonprolonged storage (≤36 months)	898 (85.4)	99.6%	98.9–99.9	<0.001	849	45	4
Specimens with prolonged storage (>36 months)	152 (14.4)	95.4%	90.7–98.1		140	5	7
Unknown	2 (0.2)	–	–	–	2	0	0

Abbreviations: CI, confidence interval; MSI, microsatellite instability; MSS, microsatellite stable; NBF, neutral buffered formalin.

### 3.2 Determination of MSI status using tumor samples alone

MSI status could be determined based on QMVR without using normal controls in 994 (94.5%) of 1052 cases. Normal tissue and blood samples were used to determine the MSI status in 25 and 27 patients, respectively. Furthermore, both normal tissue and blood samples were used to determine the MSI status in six patients. Notably, for cases in which only tumor samples were used to determine the MSI status, the proportion of MSI‐high cases (29 [42.0%] of 50) was statistically significantly lower than that of MSS cases (956 [96.5%] of 991) (*p* < 0.001).

### 3.3 TAT

**FIGURE 2:**
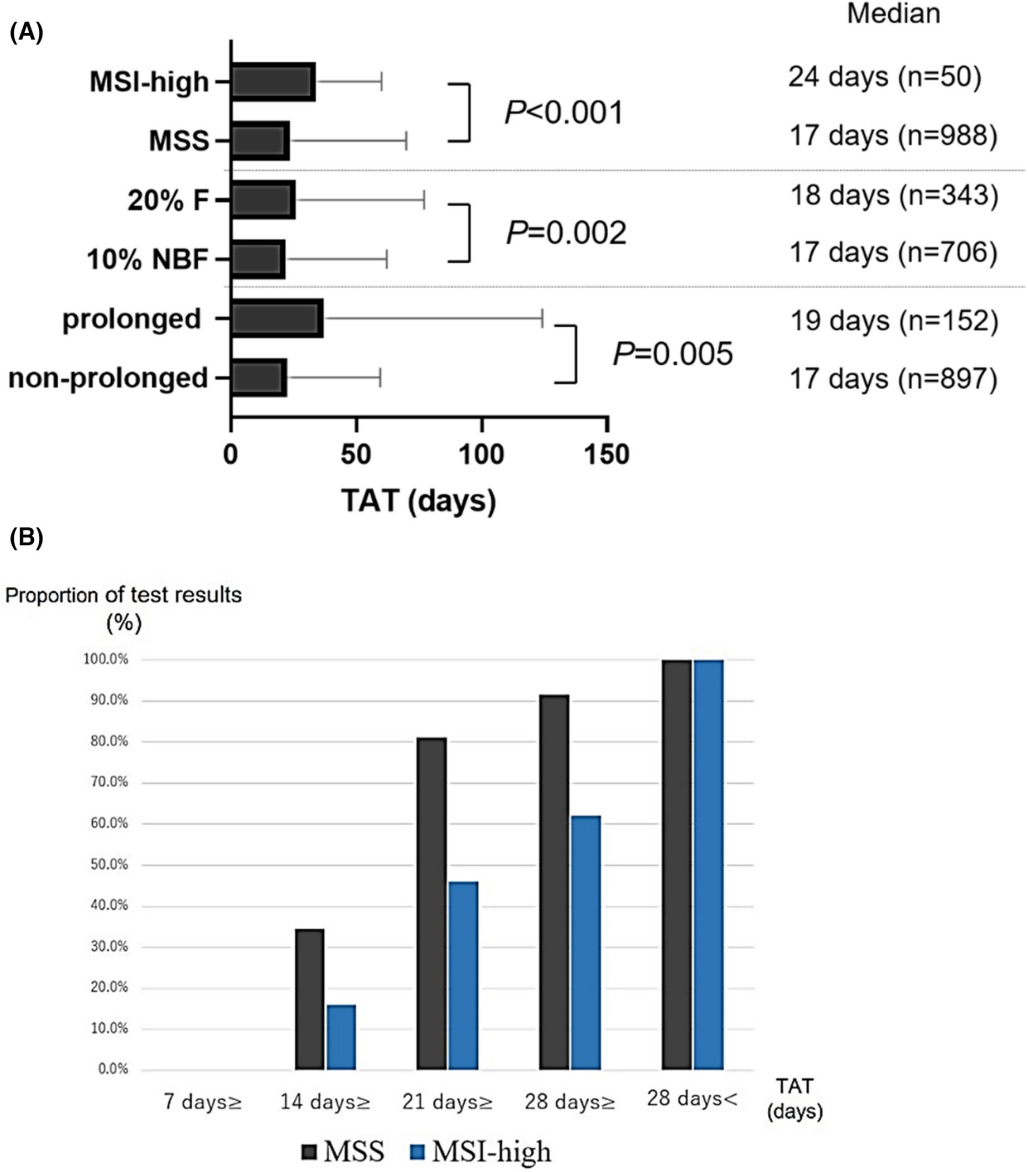
Difference in TAT. (A) Comparison of TAT between MSS and MSI‐high patients (specimens fixed with 20% NBF and 10% NBF and prolonged and nonprolonged archival specimens). (B) The bars indicate the percentages of cases with TAT of within 7, 14, 21, and 28 days and > 28 days in patients with MSS (gray) and MSI‐high (light blue) tumors, respectively. F, formalin; MSI, microsatellite instability; MSS, microsatellite stable; NBF, neutral buffered formalin; TAT, turnaround time.

The median TAT was 17 and 24 days in MSS and MSI‐high cases, respectively (*p* < 0.001) (Figure 2A). Statistically significant differences in TAT were noted between MSS and MSI‐high cases (*p* < 0.001). The TAT was within 7, 14, 21, and 28 days in 0 (0.0%), 346 (34.6%), 812 (81.2%), and 915 (91.5%) patients with MSS tumors and in 0 (0.0%), 8 (16.0%), 23 (46.0%), and 31 (62.0%) patients with MSI‐high tumors, respectively (Figure 2B). Further, 20% NBF and overfixation can lead to DNA degradation. Retesting because of DNA degradation can prolong the TAT. The TAT of specimens fixed with 20% NBF or specimens with prolonged storage was statistically significantly longer than that of specimens fixed with 10% NBF or those with nonprolonged storage (*p* = 0.002 and 0.005, respectively) (Figure 2A). The proportion of specimens with degraded DNA was higher in MSI‐high cases (*n* = 10 [20.0%]) than in MSS cases (*n* = 41 [4.1%]). However, the statistically significant differences in TAT between MSS and MSI‐high cases were maintained after excluding these 51 cases (*p* < 0.001).

### 3.4 Characteristics of patients with MSI‐high tumors treated with ICIs

Of the 50 patients with MSI‐high tumors, 24 received ICI monotherapy or combination therapy before the cutoff date. Patients with MSI‐high tumors did not receive ICIs due to the following reasons: early stage or resectable tumors (*n* = 12), ongoing therapy (*n* = 7), death (*n* = 5), patient refusal (*n* = 1), and autoimmune disease (*n* = 1) (Figure S1). The median age of these patients was 56 (range: 35–84) years. The origin of the primary tumors was as follows: CRC (*n* = 8 [33.3%]), non‐CRC gastrointestinal malignancy (*n* = 4 [16.7%]), endometrial cancer (*n* = 7 [29.2%]), and others (*n* = 5 [20.8%]). Further, 20 (83.3%) patients received monotherapy (pembrolizumab, *n* = 19; nivolumab, *n* = 1), and four (16.7%) patients with metastatic CRC (mCRC) were treated with nivolumab plus ipilimumab therapy. Only one patient did not undergo prior treatment. In total, seven (29.2%) and 16 (66.7%) patients received one or ≥two previous treatment regimens, respectively (Table S1).

### 3.5 OS, PFS, ORR, and DCR

**FIGURE 3:**
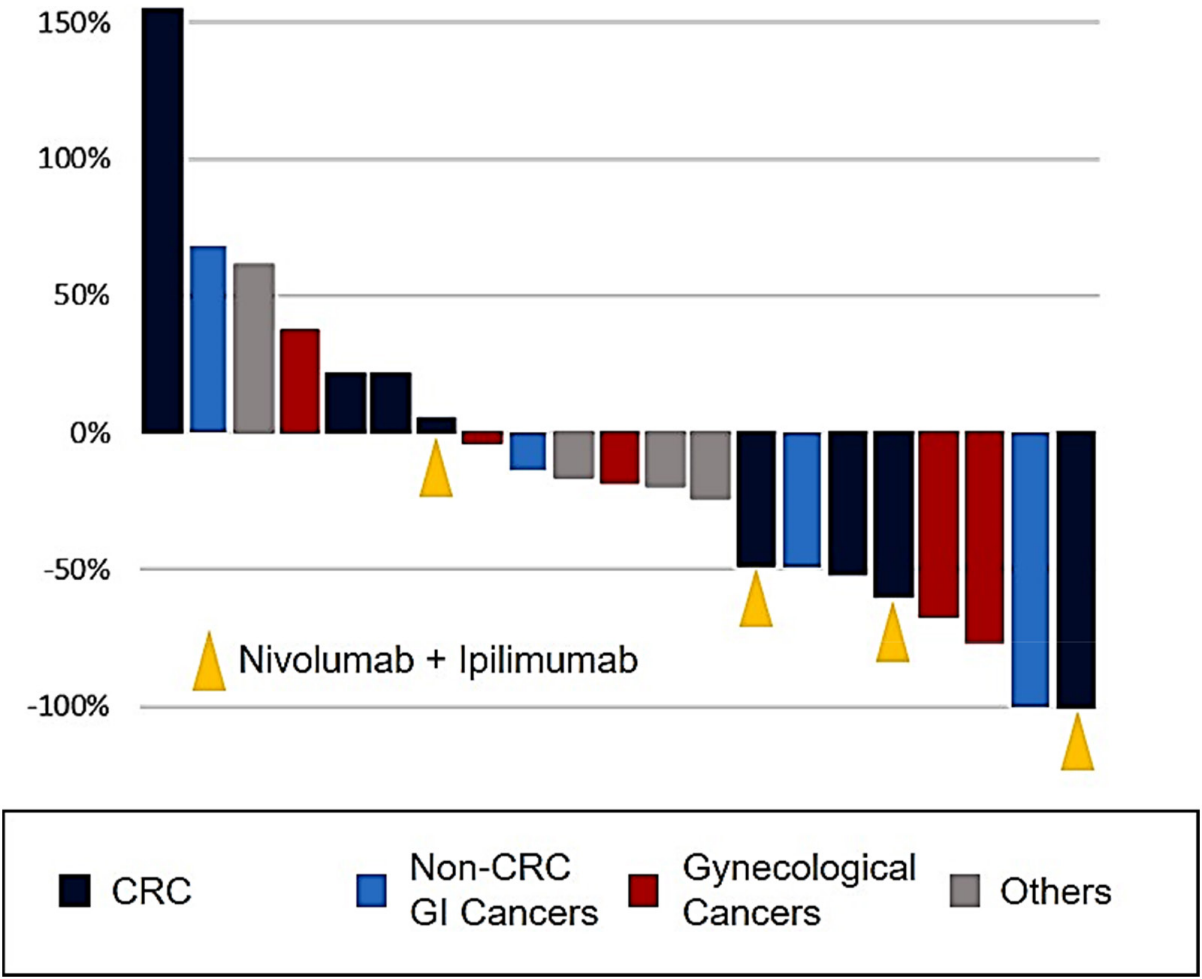
Changes in tumor diameter in MSI‐high patients (*n* = 24). CRC, colorectal cancer; GI, gastrointestinal; MSI, microsatellite instability.

On the day of analysis, 18 (75.0%) patients presented with disease progression, and seven (29.2%) died. The median PFS and OS was 5.1 (95% CI: 1.6–NA) and 23.8 months (95% CI: 6.1–NA), respectively (Figure S2A, B). The ORR and DCR in 21 patients with target lesions were 38.1% (95% CI: 18.1–61.6) and 66.7% (95% CI: 43.0–85.4), respectively (Table 2). Among them, 14 (66.7%) patients presented with a median tumor shrinkage rate of 18.0% from the baseline (range: −157.9% to 100.0%) (Figure 3).

**TABLE 2:** Objective response rate

	MSI‐high cases (*n* = 21)
	*n*(%)
Complete response	1(4.8)
Partial response	7(33.3)
Stable disease	6(28.6)
Progressive disease	7(33.3)
Overall response rate (95% CI)	38.1%(18.1–61.6)
Disease control rate (95% CI)	66.7%(43.0–85.4)

Abbreviations: CI, confidence interval; MSI, microsatellite instability.

### 3.6 Genetic consultation for patients with MSI‐high solid tumors

**FIGURE 4:**
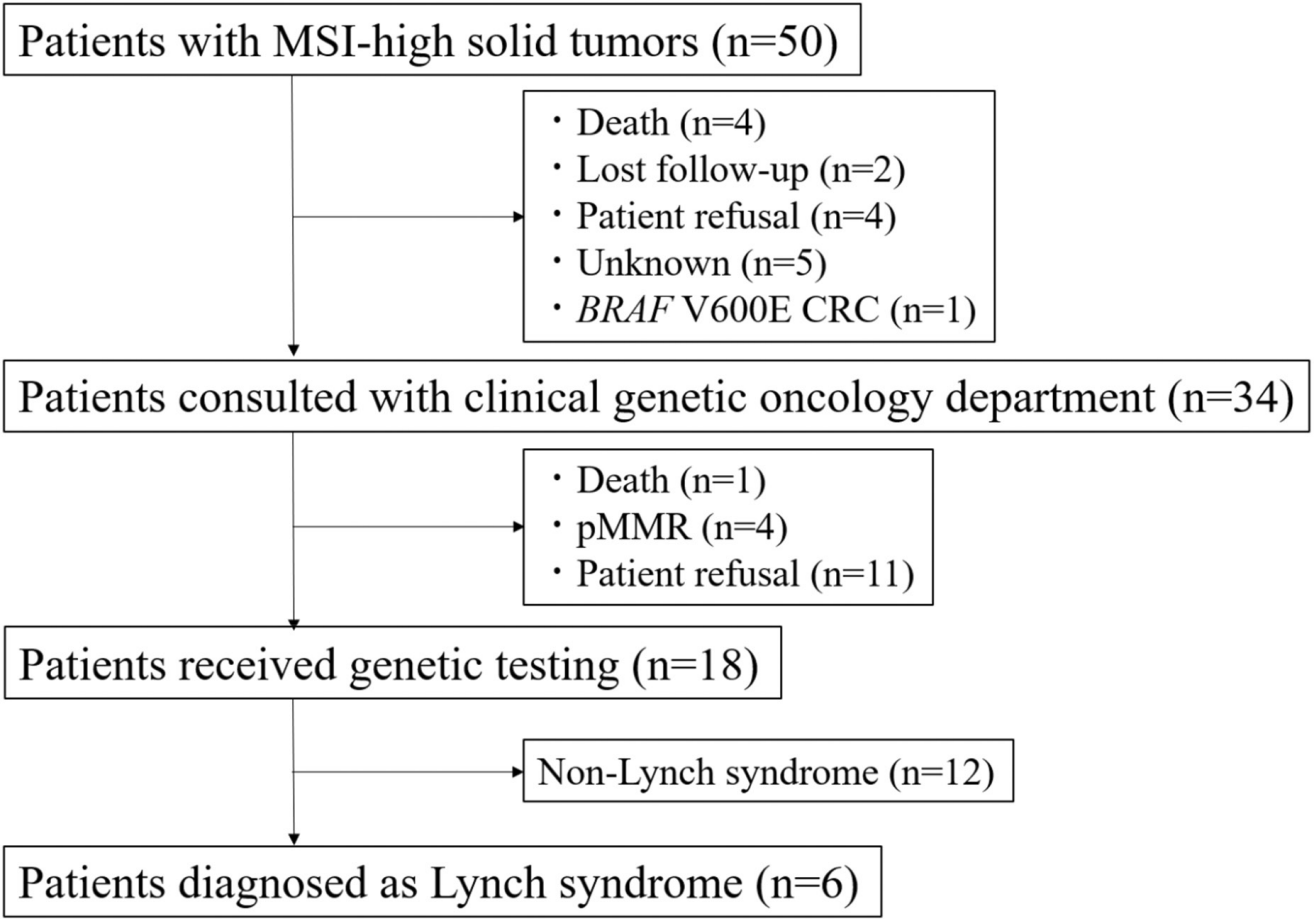
Flowchart of patients with MSI‐high tumors. BRAF, Raf murine sarcoma viral oncogene homolog B; CRC, colorectal cancer; MSI, microsatellite instability; pMMR, proficient mismatch repair.

Of the 50 patients with MSI‐high tumors, 34 were referred to the Department of Clinical Genetic Oncology (Figure 4). Death or loss to follow‐up (*n* = 6) was the most common reason for no consultation. Of these 34 patients, 18 underwent genetic testing. Six patients were diagnosed with Lynch syndrome. Among them, one patient was known to have Lynch syndrome; three were newly diagnosed after tumor MSI testing, which is used as a screening tool for Lynch syndrome; and two were newly diagnosed after tumor MSI testing for determining the indication of ICI. Of the remaining 14 patients who did not undergo genetic testing, four presented with pMMR and one died after the initial consultation. The most common reason for not conducting genetic testing was patient refusal (*n* = 11).

## 4 DISCUSSION

MSI testing via PCR‐based methods with a mononucleotide panel has been successfully used in clinical practice for assessing different types of tumors using specimens with prolonged storage or small biopsy specimens. The clinical outcomes of patients with MSI‐high tumors treated with ICIs were comparable to those reported in previous trials.^9^, ^11^ Our real‐world study revealed that MSI testing showed a lower success rate in patients with overfixed specimens and longer TAT in MSI‐high cases; moreover, it indicated the inadequate awareness of MSI testing as a screening tool for Lynch syndrome.

The overall success rate of MSI testing in this study (99.0%) was similar to that reported in previous large‐scale real‐world studies (99.1%) in Japan.^13^ In particular, the success rate in patients with overfixed specimens was low (>95%). Further, pH, formalin concentration, and fixation time can affect DNA degradation.^17^ Specimen quality was found to be associated with prolonged TAT. MSI testing was introduced in 2019 at our hospital after the implementation of a standard protocol for genomic testing of pathological specimens. Therefore, only overfixed and archived specimens were available at that time. Ideally, the optimal specimen for MSI testing would improve the success rate and TAT. However, it is challenging to strictly comply with this recommendation in daily practice. Recently, in addition to the use of several biomarkers for patient selection, such as HER2, PD‐L1, EGFR, and KRAS, personalized therapy with next‐generation sequencing (NGS)‐based multigene panels is increasingly being used in several countries,^18^ and the demand for optimal specimens is increasing. DNA degradation can potentially lead to amplification failure. However, if the specimens are well amplified, MSI testing can be performed. In our study, most specimens that were fixed with 20% NBF and those with prolonged storage could be successfully subjected to MSI testing. Furthermore, MSI‐high tumors belong to a low‐incident tumor subset (<5%). Thus, testing based on high‐quality specimens is less likely to be prioritized than other biomarker tests. NGS‐based testing can identify the MSI status and comprehensive genome profile and provide a useful alternative when the amount of specimen is low.^19^, ^20^, ^21^, ^22^ The development of NGS‐based MSI testing can improve precision oncology. Furthermore, the plasma‐based detection of MSI status can help overcome limitations in specimen amount.^23^ The application of NGS‐based comprehensive genome profiling including MSI status (Foundation One CDx®) was approved in Japan in June 2021. MSI testing using NGS‐based methods could be an alternative method. However, the NGS‐based method cannot yet replace the PCR‐based MSI testing. The use of NGS‐based genome testing in previously untreated patients has not been approved and is limited to patients with metastatic disease. Thus, we believe that the demand for PCR‐based methods will continue in the near future.

Shortening the TAT for MSI testing is an urgent issue that must be addressed in future studies on precision oncology. Recently, the KN‐177 trial revealed that the efficacy of pembrolizumab alone was superior to that of standard chemotherapy when used as the first‐line treatment for patients with MSI‐high mCRC.^24^ Delayed diagnosis may significantly affect its treatment. Compared with MSS cases, a higher proportion of matched normal samples (tissue or blood samples) were required for MSI‐high cases, and this is the main reason for the prolonged TAT. Bando et al. revealed that MSI testing based on QMVR using tumor samples alone showed a higher concordance rate than the conventional method with paired normal samples in a Japanese cohort.^15^ However, owing to the low prevalence of MSI‐high mCRC cases, only 11 cases were included in their study.^15^ Thus, the application of MSI testing based on QMVR of mononucleotide markers in MSI‐high cases must be further investigated. MMR–IHC analysis is an effective and feasible alternative method for shortening the TAT in MSI‐high cases. Patients with dMMR tumors who were diagnosed using local MMR–IHC analysis were enrolled in pivotal clinical trials, such as the KN‐158, KN‐164, and KN‐177 studies.^9^, ^11^, ^24^ However, MMR–IHC analysis is not universally available.^25^ In September 2022, MMR–IHC was just approved in Japan. MMR–IHC analysis could address the limitations of PCR‐based MSI testing, particularly TAT.^26^ However, PCR‐based MSI testing might be useful in cases that cannot be diagnosed via MMR–IHC analysis alone.^27^ Based on demand, the combination of MSI testing and MMR–IHC analysis could be feasible in clinical practice.

MSI testing is commonly conducted to determine ICI indications for most cases, and it could also be used as a screening tool for Lynch syndrome. However, of the 50 patients with MSI‐high tumors in our study, 16 did not consult hereditary tumor experts because of death or loss to follow‐up after disease progression (*n* = 6). In addition, 11 of 34 patients who were referred to the Clinical Genetic Department refused to undergo germline tests. This may be due to the procedural cost. The National Insurance System does not cover genetic testing for cancer prevention. The genetic testing result has no effect on the therapeutic strategy itself, and some patients believed that genetic testing was not cost‐effective. To improve the diagnosis of Lynch syndrome via MSI testing, its cost should be reduced. However, the medical costs in the aging population of Japan are continually rising, thereby decreasing the possibility of further increase in cost. In addition, due to the low rate of genetic testing, there may be no reduction in the cost of this procedure. Therefore, it is less likely to observe significant increases in the near future. A steady attempt to explain the need for MSI testing over time is important and is the most feasible option. Delayed MSI testing or long TAT may have affected the results. In addition, the timing of MSI testing is key from the viewpoint of Lynch syndrome screening. Patients with severe cancer‐associated symptoms must pay considerable attention to future cancer risk. Early MSI testing could enhance the accessibility of genetic testing or counseling. Nevertheless, we could not determine the reason for no consultation in the medical records of five cases; moreover, we might have paid inadequate attention to familial or hereditary cancers in daily practice. More clinicians could potentially provide the opportunity for patients with MSI‐high tumors to undergo genetic testing and counseling. Further understanding of cancer prevention and health care among patients' relatives is important to facilitate the successful growth of genomic medicine.

Our study has several limitations. First, the number of patients with MSI‐high tumors was relatively small. Owing to the low incidence of MSI‐high tumors and the fact that the study was performed at a single institution, most types of tumors, including gastric, urothelial, and renal cell lesions, which are indicated for ICIs regardless of the MSI status, were eliminated from MSI testing. There were some discrepancies in the specimen quality for MSS and MSI‐high cases. Due to the small number of cases, we could not eliminate the effects of confounding factors on the TAT. Second, this study was conducted at a single institution in Japan. The fixation protocol was only used at our institution, and specimen selection was based on the discretion of the attending pathologists. Third, we outsourced the MSI testing, and the TAT included the shipping time from our laboratory to the inspection company as well as reporting delays. Thus, in this study, the TAT of MSI testing was slightly longer than that of in‐house testing. However, the TAT differed according to the MSI status.

In conclusion, more than 1000 real‐world studies have reported the versatility and reliability of MSI testing using different types of tumor samples in clinical practice. However, TAT may be affected by specimen quality and MSI status. Prolonged TAT can delay treatment in patients with MSI‐high tumors. Increasing the number of methods used for determining MSI status is a potential solution for issues such as limited availability of optimal specimens and TAT. Furthermore, awareness regarding the importance of hereditary tumors among clinicians is important for the successful growth of precision oncology.

## AUTHOR CONTRIBUTIONS

**Izuma Nakayama:** Conceptualization (lead); data curation (lead); formal analysis (lead); investigation (lead); methodology (lead); project administration (lead); writing – original draft (lead); writing – review and editing (lead). **Eiji Shinozaki:** Conceptualization (equal); project administration (supporting); supervision (supporting); writing – review and editing (supporting). **Hiroshi Kawachi:** Conceptualization (supporting); data curation (supporting); investigation (supporting); writing – review and editing (supporting). **Takashi Sasaki:** Investigation (supporting); writing – review and editing (supporting). **Mayu Yunokawa:** Investigation (supporting). **Junichi Tomomatsu:** Investigation (supporting). **Takeshi Yuasa:** Investigation (supporting); writing – review and editing (supporting). **Satoru Kitazono:** Investigation (supporting). **Kokoro Kobayashi:** Investigation (supporting). **Keiko Hayakawa:** Investigation (supporting). **Arisa Ueki:** Data curation (supporting); investigation (supporting); writing – review and editing (supporting). **Shunji Takahashi:** Project administration (supporting); supervision (supporting). **Kensei Yamaguchi:** Project administration (lead); supervision (lead).

## CONFLICT OF INTEREST

The authors have no conflict of interest to declare.

## ETHICAL APPROVAL

This study was approved by the ethics committee of the Cancer Institute Hospital of the Japanese Foundation for Cancer Research (JFCR) in Tokyo, Japan (approval no. 2020–1229) and was conducted in accordance with the tenets of the Declaration of Helsinki (1964) and its later amendments.

## Supporting information


Figure S1.

Figure S2.

**Table S1.** *n*

## Data Availability

The data supporting the findings of this study are available from the corresponding author upon reasonable request.
