# Supplementary figures and images for: Implementation of microsatellite instability testing for the assessment of solid tumors in clinical practice
Source: Cancer Med. 2022 Dec 26;12(7):7932–40. doi: 10.1002/cam4.5569 (PMC10134335; doi:10.1002/cam4.5569)

## Slide 1
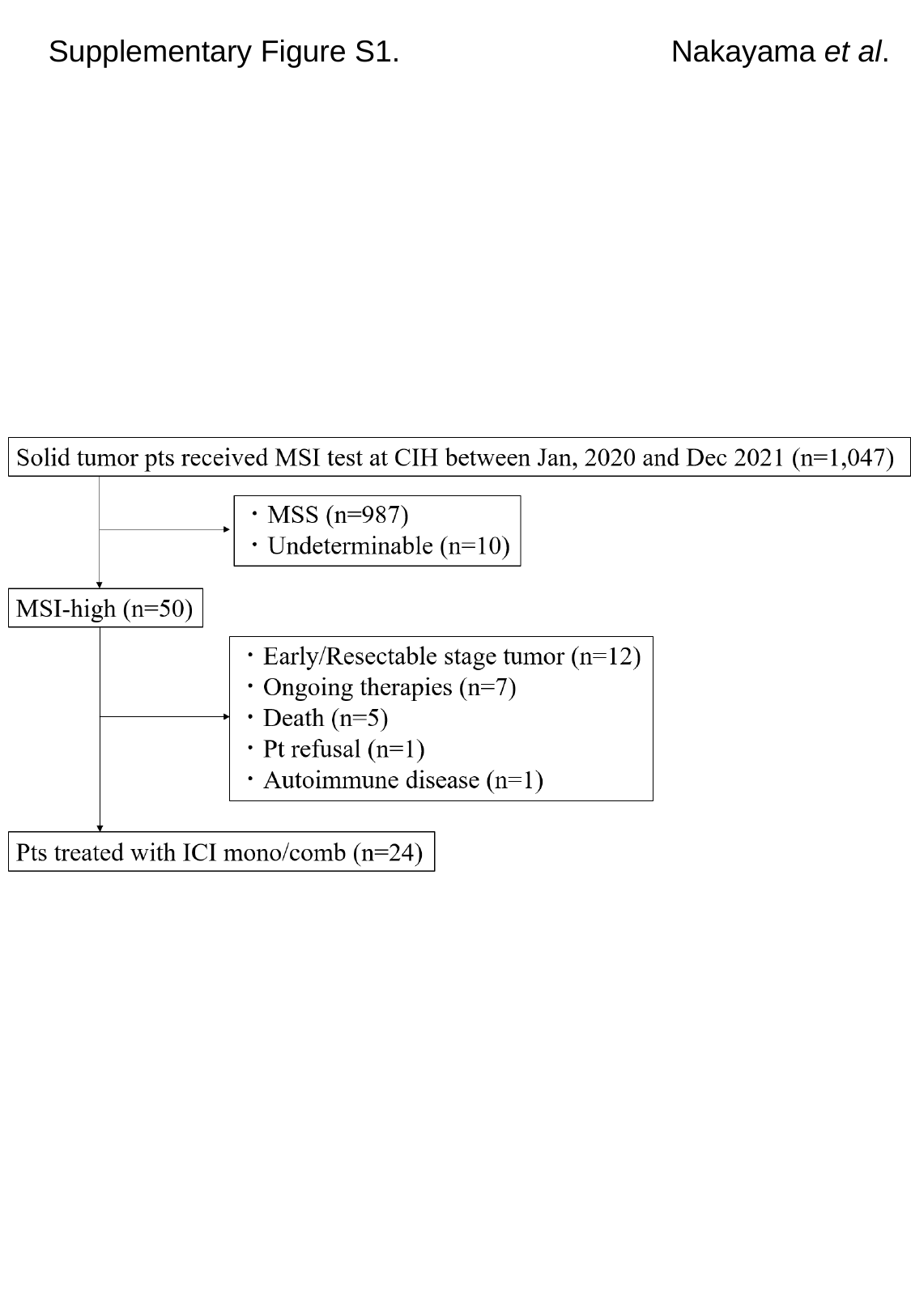

Supplementary Figure S1.
Nakayama et al.

Supplement: Supplementary file 1 — Figure S1. [file CAM4-12-7932-s001.pptx]

## Slide 1
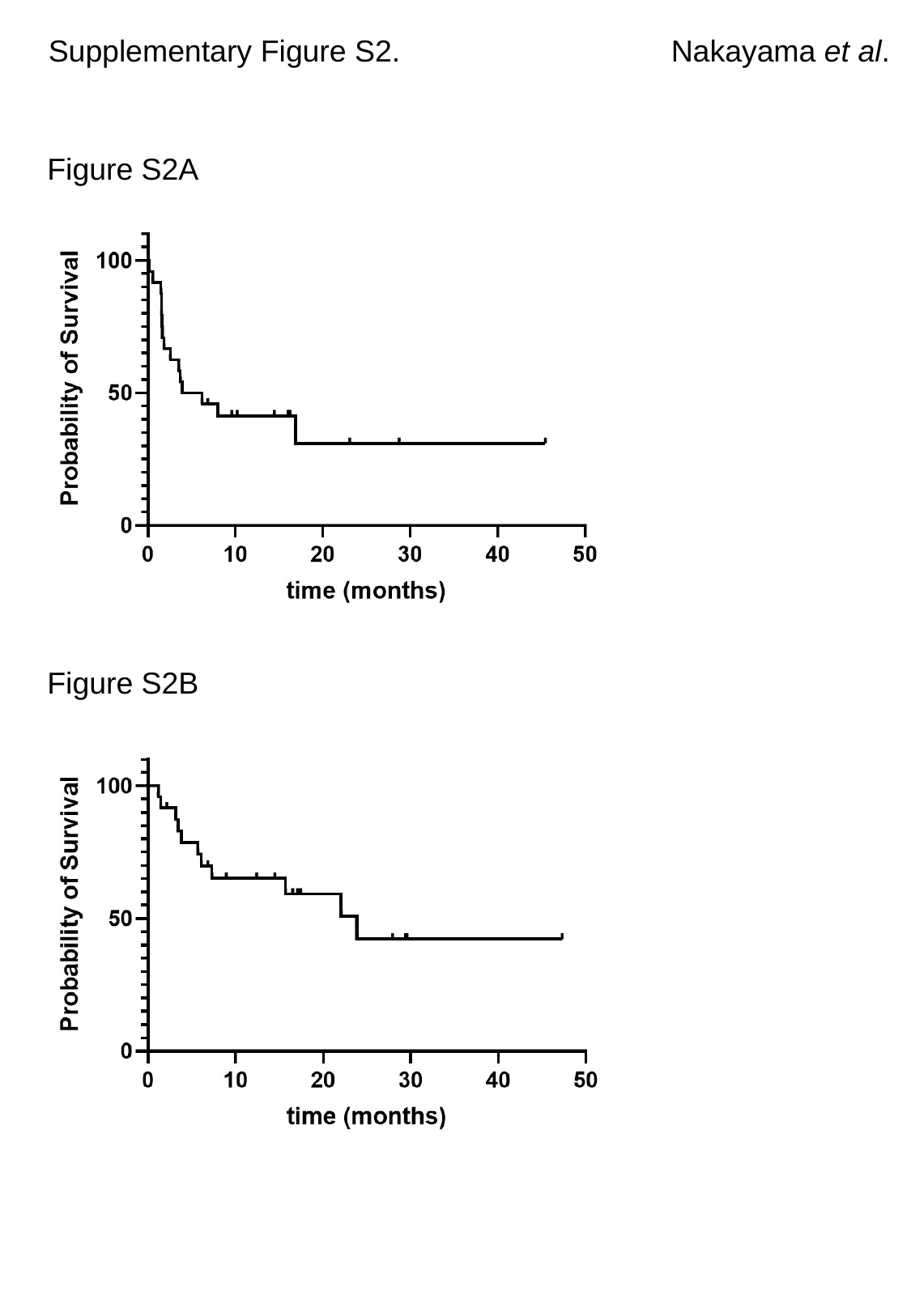

Supplementary Figure S2.
Nakayama et al.
Figure S2A
Figure S2B

Supplement: Supplementary file 2 — Figure S2. [file CAM4-12-7932-s003.pptx]
